# Diffusional-displacive transformation enables formation of long-period stacking order in magnesium

**DOI:** 10.1038/s41598-017-04343-y

**Published:** 2017-06-22

**Authors:** Jin-Kyung Kim, Lei Jin, Stefanie Sandlöbes, Dierk Raabe

**Affiliations:** 1 Max-Planck-Institut für Eisenforschung GmbH, 40237 Düsseldorf, Germany; 2 Ernst Ruska-Centre for Microscopy and Spectroscopy with Electrons (ER-C) and Peter Grünberg Institute (PGI-5), Research Centre Jülich, 52425 Jülich, Germany; 3 Institute of Physical Metallurgy and Metal Physics, RWTH Aachen University, 52056 Aachen, Germany; 4 Graduate Institute of Ferrous Technology, Pohang University of Science and Technology, 77 Cheongam-Ro, Pohang, 37673 South Korea

## Abstract

Mg is the most important lightweight engineering alloy enabling future weight-reduced and fuel-saving engineering solutions. Yet, Mg is soft. Long-period stacking ordered (LPSO) structures in Mg alloys have unique crystal structures, characterized by both complex chemical and stacking order. They are essential for strengthening of Mg alloys. The formation mechanism of these LPSO structures is still under discussion. Here we report that Y/Zn enriched Guinier-Preston (GP) zones observed in a lean Mg-Y-Zn model alloy are precursors of early stage LPSO structures. We provide evidence of a new type of phase transformation mechanism which comprises the diffusional formation of Y/Zn enriched GP zones and their subsequent shear transformation into LPSO building blocks. The mechanism constitutes a new type of coupled diffusional-displacive phase formation sequence which may also be applicable to other alloy systems.

## Introduction

Mg and Mg based alloys are key materials for future lightweight applications due to their low mass density^1^. The main drawbacks of Mg and most commercial Mg alloys are intrinsic brittleness and low strength. Complex chemical and structural ordered zones referred to as long-period-stacking-ordered (LPSO) phases^2–4^ have enabled considerable strengthening of Mg alloys together with decent ductility compared to conventional Mg alloys^5–7^. Generally, LPSO structures are formed in Mg-transition metal-rare earth based systems, with the Mg-Y-Zn system being the most extensively studied one. LPSO structures have the same {0001} basal plane as Mg while their stacking periodicity is modified along the c-axis compared to that of Mg. LPSO phases include polytypes expressed as 10H, 14H, 18R and 24R^2, 4, 8^. Here, the integer indicates the number of atomic layers in one period and the letters H and R stand for hexagonal and rhombohedral symmetry, respectively^9^. LPSO structures consist of Y/Zn enriched building blocks that have a local fcc stacking sequence on the close packed planes^10^. The local fcc stacking sequence of the LPSO building blocks is due to the existence of bounding Shockley partial dislocations at each end of the building blocks. The formation of Zn_6_Y_8_ clusters of L1_2_ type has been often observed mainly in the building blocks of highly alloyed Mg-Y-Zn systems^2, 11, 12^. Hence, LPSO structures are both chemically and stacking ordered. Here we report that Y/Zn enriched GP zones observed in a lean Mg-Y-Zn model alloy are precursors of early stage LPSO structures.

Guinier-Preston (GP) zones, named after the first observations by Guinier^13^ and Preston^14^, have enabled strengthening of aerospace alloys such as Al and Ti. Contrary to LPSO structures exhibiting both chemical and stacking order, Guinier-Preston (GP) zones only exhibit chemical ordering, maintaining the crystal structure of metallic alloys. During the past decades, extensive research has focused on the structural-chemical evolution and resulting strengthening mechanisms of GP zones in Al alloys using X-ray diffraction and electron microscopy^15–21^. Two kinds of GP zones, GP (1) zones and GP (2) zones, have been reported in Al-Cu alloys^20, 21^. Single Cu-rich layers are called GP (1) zones whereas GP (2) zones show two Cu-rich layers separated by three Al layers^21^. GP zones are metastable phases which form during heat treatment processes as a preliminary stage of the formation of metastable and stable precipitates upon prolonged heat treatment processes. Despite the progress in understanding the structures of GP zones in various alloy systems, their structural evolution mechanisms still remain unclear.

During the past decade, GP zones in Mg alloys have been studied^22–26^. Monolayer GP zones on the {0001} basal plane of the Mg matrix have been observed in an Mg-RE-Zn-Zr alloy^22^. The GP zones have been found to be enriched in rare earth elements and Zn atoms^22^. These monolayer GP zones have also been observed in an Mg-Ca-Zn alloy^23^. Another types of basal GP zones composed of two (Gd, Zn)-rich layers, separated by a single Mg layer, have been reported in Mg-Gd-Zn alloys^24, 25^. Bilayer GP zones forming on two consecutive basal planes of the Mg matrix have been also reported in an Mg-Y-Zn-Nd alloy^26^. Consequently, the structure and chemical ordering of GP zones in Mg alloy are expected to be highly composition-dependent.

Despite the high importance of LPSO structures as a key mechanism for strengthening of Mg alloys, the formation mechanisms of LPSO structures are still under discussion. LPSO structures are considered to develop from a single building block^27^. A recent study on the formation of LPSO structures in a highly alloyed Mg-Y-Zn system using small-angle X-ray scattering suggests a hierarchical phase transformation sequence of atomic clustering, followed by cluster motion and the formation of stacking faults to form LPSO structures^28^. In the present study, we aim at identifying the formation mechanisms of LPSO structures by direct microstructural investigations using aberration-corrected high angle annular dark field scanning transmission electron microscopy (HAADF-STEM) and atomic-scale energy dispersive x-ray spectroscopy (EDXS) mapping. We focus on a lean alloyed Mg-1Y-0.5Zn (wt. %) system to observe the early stages of LPSO structure formation during heat treatments. Our study provides direct evidence of the phase transformation from Y/Zn enriched GP zones to LPSO building blocks indicating that Y/Zn enriched GP zones are a pre-stage of LPSO structures. The formation of these LPSO structures represents a new type of coupled diffusional-displacive phase formation sequence which may also be applicable to other alloy systems.

## Results

### Structural evolution from GP zone to LPSO building block

**Figure 1:**
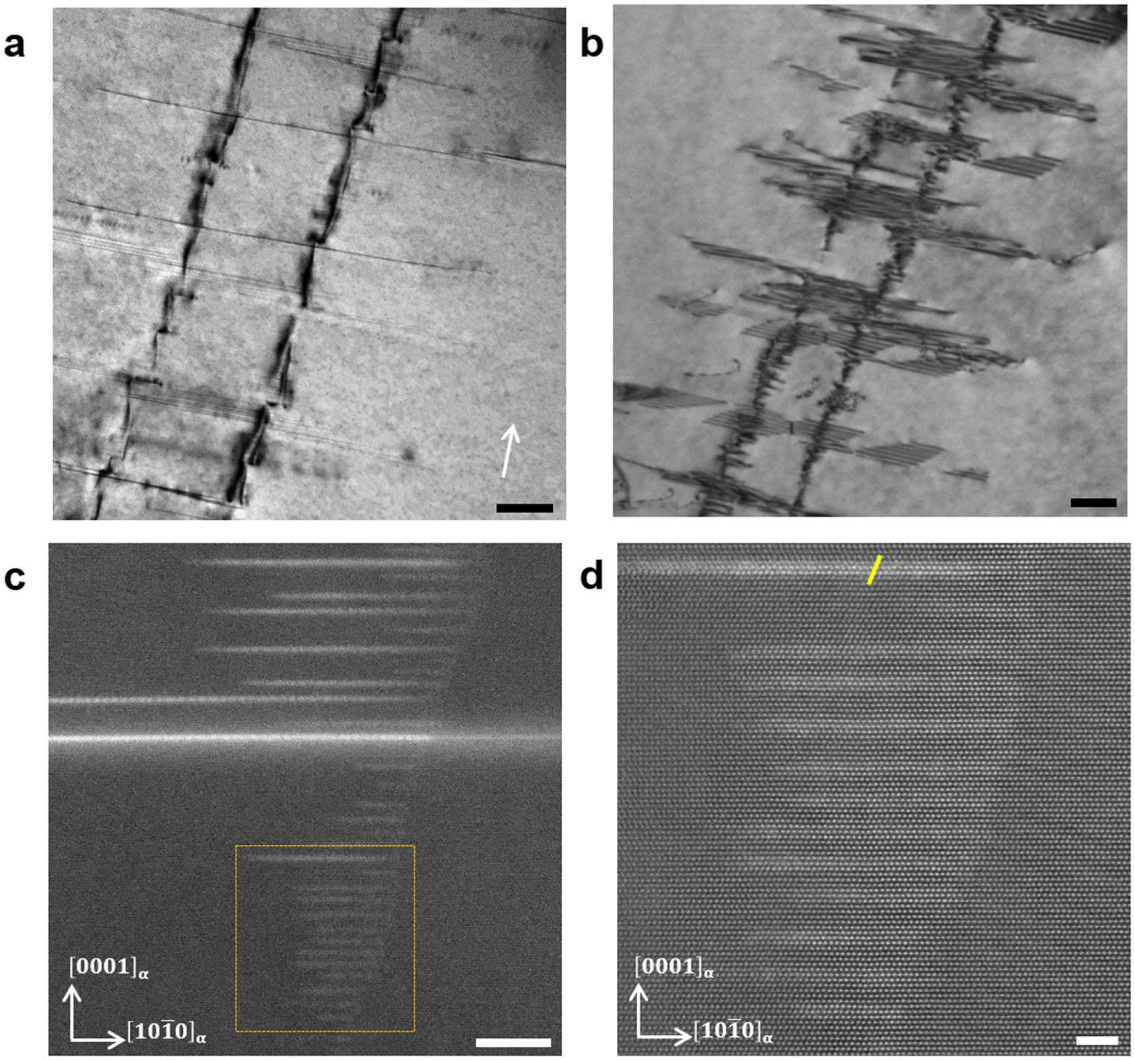
GP zones and LPSO building blocks in solution-annealed Mg-1Y-0.5Zn alloy. (**a**) TEM bright field image under two-beam condition with g = {0002} for Mg-1Y-0.5Zn alloy after solution annealing for 10 h at 500 °C followed by air cooling. Two main microstructural features can be seen in the Mg matrix: long plate-type defects along the basal plane and between the long plate-type defects strong strain contrast along the hcp c-axis (parallel to the white arrow). Beam direction is near $[1\bar{2}10]$. Scale bar width is 200 nm. (**b**) TEM bright field image of the same region as (**a**) under two-beam condition with g = $(10\bar{1}1)$, which reveals a more inclined view of the basal plane. The long plate-type defects along the basal plane in (**a**) show the contrast of stacking fault fringes together with some Shockley partial dislocations. The strong strain contrast in (**a**) shows the thin line contrast without the contrast of stacking fault fringes. Scale bar width is 200 nm. (**c**) HAADF-STEM image shows long plate-type defects and very thin defects with bright contrast, which indicates that the defects are enriched in Y/Zn atoms. Beam direction is $[1\bar{2}10]$. Scale bar width is 10 nm. (**d**) Enlarged HAADF-STEM image of the region marked by an orange square in (**c**). The uppermost basal plane defect with bright contrast shows a local fcc stacking sequence being structurally identical with LPSO building blocks. The yellow line illustrates the stacking sequence of the LPSO building block. The other thin basal defects are hcp-GP zones enriched in Y/Zn atoms. Beam direction is $[1\bar{2}10]$. Scale bar width is 2 nm.

Figure 1a shows a TEM bright field image under two-beam condition with g = {0002} for Mg-1Y-0.5Zn alloy after solution annealing for 10 h at 500 °C followed by air cooling, revealing two main microstructural features in the Mg matrix: long plate-type defects along the basal plane and between the long plate-type defects strong strain contrast along the hcp c-axis (parallel to the white arrow in Fig. 1a). Figure 1b shows a TEM BF micrograph of the same region as Fig. 1a under two-beam condition with g = $(10\bar{1}1)$, which reveals a more inclined view of the basal plane. The long plate-type defects along the basal plane in Fig. 1a show the contrast of stacking fault fringes together with some Shockley partial dislocations in Fig. 1b. Further, the strong strain contrast in Fig. 1a shows thin line contrast without the contrast of stacking fault fringes in Fig. 1b. The HAADF-STEM image in Fig. 1c presents the same microstructural features as Fig. 1a. HAADF-STEM gives contrast proportional to Z^p^ where Z is the atomic number and p is a coefficient in the range of 1.4–2. The plate-type defects on the basal plane shown in Fig. 1a exhibit bright contrast in the HAADF imaging mode, which indicates that the defects are enriched in Y and Zn. In addition, very thin (few atomic layers thick) defects with bright contrast are present between the long plate-type defects. These thin defects are assumed to have caused the strong strain contrast along the hcp c-axis shown in Fig. 1a. Figure 1d shows an enlarged image of the region marked by an orange square in Fig. 1c. The uppermost basal plane defect exhibits stronger bright contrast than the other basal plane defects and shows a local fcc stacking sequence being structurally identical to LPSO building blocks of Mg-Y-Zn alloys^27^. The other thin basal plane defects in Fig. 1c also exhibit bright contrast, however, with a Z-contrast intensity lower than the LPSO building block. These thin basal plane defects have an hcp structure with an ABAB stacking sequence and are therefore identified as GP zones enriched in Y/Zn atoms. The thickness of the GP zones is in the range of 2–4 Mg basal plane atomic layers while the length of the GP zones is in the range of 5–15 nm.

**Figure 2:**
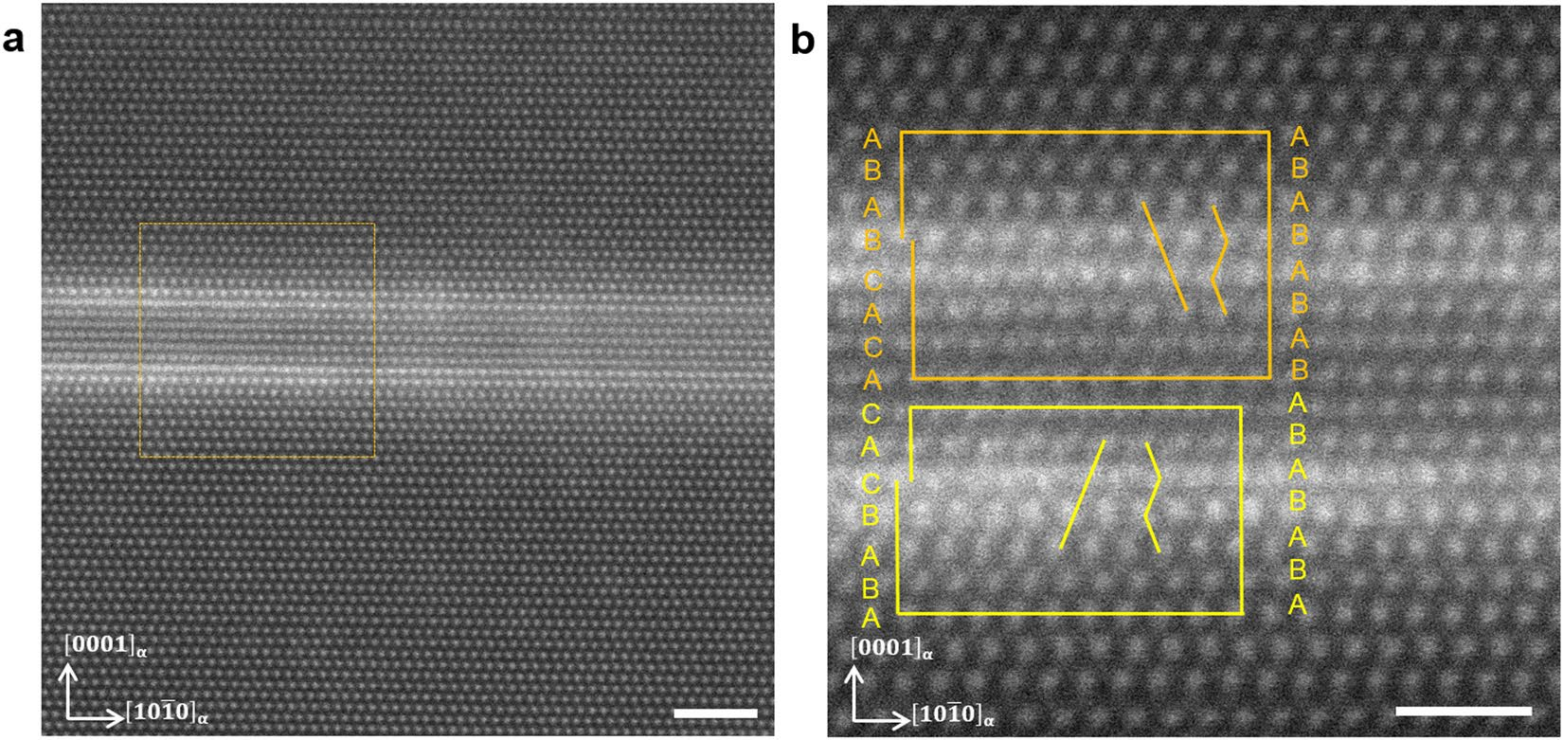
Structural evolution from GP zones to LPSO building blocks. Atomic scale HAADF-STEM images of the Mg-1Y-0.5Zn alloy after solution annealing for 10 h at 500 °C. Beam direction is $[1\bar{2}10]$. (**a**) Two Y/Zn enriched basal plane defects. The basal plane defects on the right side of the image show an hcp stacking sequence which changes to the local fcc stacking sequence characteristics for the LPSO building blocks on the left side of the image. Scale bar width is 2 nm. (**b**) A magnified image of the orange square region in (**a**). The upper basal plane defect (orange Burgers circuit) changes its stacking sequence from an ABAB-type hcp stacking to an ABCA and the lower basal plane defect (yellow Burgers circuit) to an ACBA fcc stacking. Scale bar width is 1 nm.

Direct evidence of the formation of LPSO building blocks from the Y/Zn enriched GP zones is presented in Fig. 2. Figure 2a shows two basal plane defects with bright contrast indicating that the regions are enriched in Y/Zn atoms. The high Z-contrast intensity of each basal plane defect is confined to two consecutive basal planes. It should be noted here that the intensity of the Z-contrast of the basal plane defects increases from the right side to the left side of the image. Further, the basal plane defects on the right side of the image show an hcp stacking sequence which changes to the local fcc stacking sequence characteristic for the LPSO building blocks on the left side of the image. Figure 2b shows a magnified image of the square region in Fig. 2a. Orange and yellow colored Burgers circuits are drawn for the upper and lower basal plane defects. The upper basal plane defect changes its stacking sequence from an ABAB-type hcp stacking to an ABCA and the lower basal plane defect to an ACBA fcc stacking. Hence, the defect regions with hcp structure are therefore identified as Y/Zn enriched GP zones and the defect regions with fcc stacking as LPSO building blocks. The observed change from hcp to fcc stacking of the basal plane defects shown in Fig. 2 indicates that basal <a> Shockley partial dislocations were generated inside the Burgers circuits and propagated to the left side of the image for both basal plane defects. The Burgers circuit analysis indeed shows that both basal plane defects exhibit $\frac{a}{3}\langle 10\bar{1}0\rangle$- type basal <a> Shockley partial dislocations with opposite signs at the transition between hcp and fcc stacking. The different Z-contrast intensity of the GP zones and the LPSO building blocks suggests that the GP zones transform to LPSO building blocks if a critical local concentration of Y/Zn is reached. The structures on the left side of the image correspond to a 14H LPSO structure composed of two LPSO building blocks separated by three Mg layers. This indicates that the stacking faults subsequently form LPSO building blocks in Mg-Y-Zn alloys. The separation distance between the GP zones is therefore assumed to play an important role for the formation of LPSO structures.

**Figure 3:**
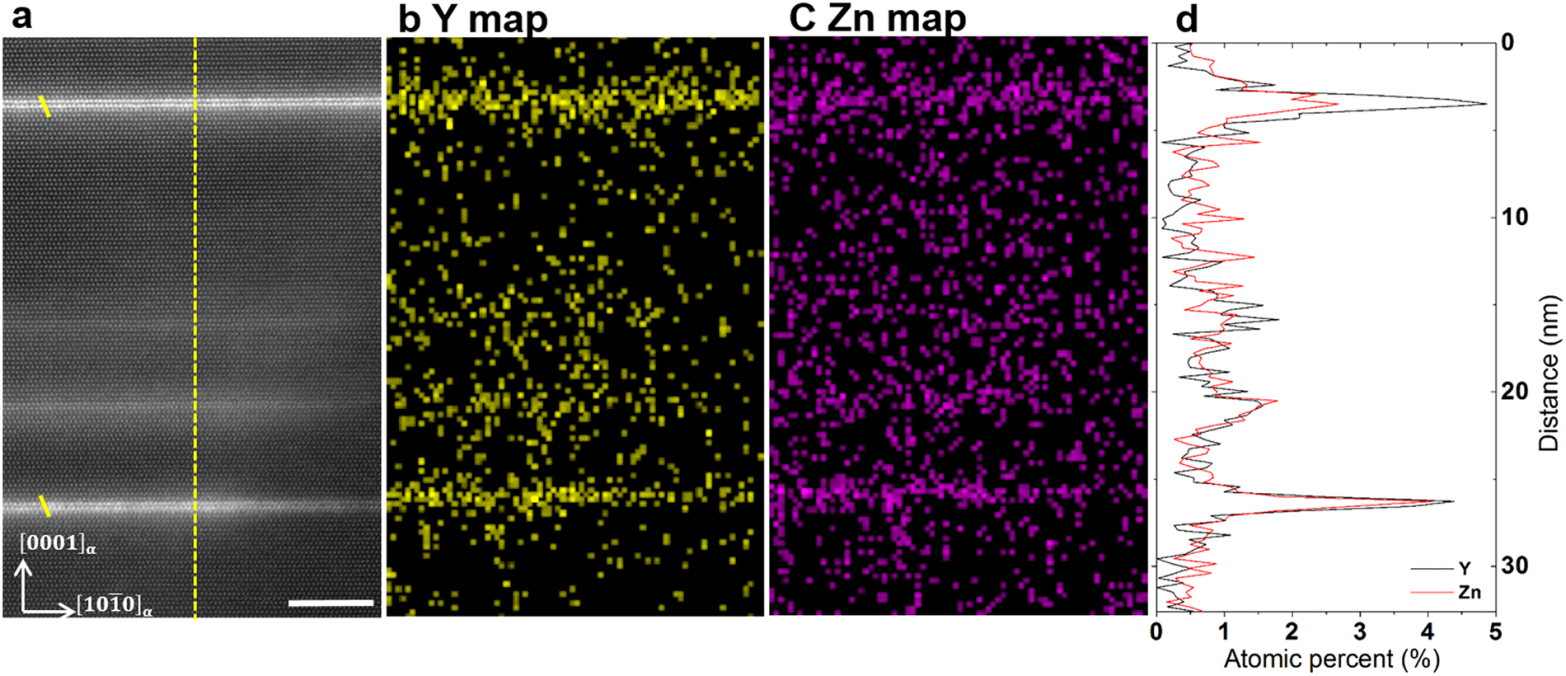
Correlative HAADF-STEM and EDXS analysis for the GP zones and LPSO building blocks. (**a**) HAADF-STEM image of the Mg-1Y-0.5Zn alloy after solution annealing for 10 h at 500 °C. Beam direction is $[1\bar{2}10]$. Four basal plane defects which are either GP zones or LPSO building blocks. The yellow lines illustrate the stacking sequence of the LPSO building blocks. Scale bar width is 5 nm. (**b**,**c**) Correlative TEM-EDXS atomic distribution maps of Y and Zn atoms. (**d**) The quantitative concentrations of Y and Zn in at. % along the line drawn in (**a**). It is evident that the LPSO building blocks are more enriched in Y/Zn atoms than the GP zones.

To quantify the observation of higher Z-contrast intensity in LPSO building blocks than in GP zones, we performed a correlative structural and chemical analysis using HAADF-STEM and TEM-EDXS as shown in Fig. 3. Figure 3a shows four basal plane defects which are either GP zones or LPSO building blocks. The Z-contrast intensity of the LPSO building block is more pronounced than that of the GP zones. This is consistent with the TEM-EDXS atomic distribution maps of Y and Zn given in Fig. 3b and c. Fig. 3d shows the quantitative concentrations of Y and Zn in at. % along the line drawn in Fig. 3a. It is evident that the LPSO building blocks are more enriched in Y/Zn atoms than the GP zones. TEM-EDXS observations in other sample regions confirm that the concentration of Y and Zn is generally above 5 at. % in LPSO building blocks while GP zones generally contain less than 5 at. % Y and Zn. We therefore assume that the local concentration of Y and Zn atoms governs the transformation from GP zones to LPSO building blocks.

### Building block clusters of long period stacking ordered structures

**Figure 4:**
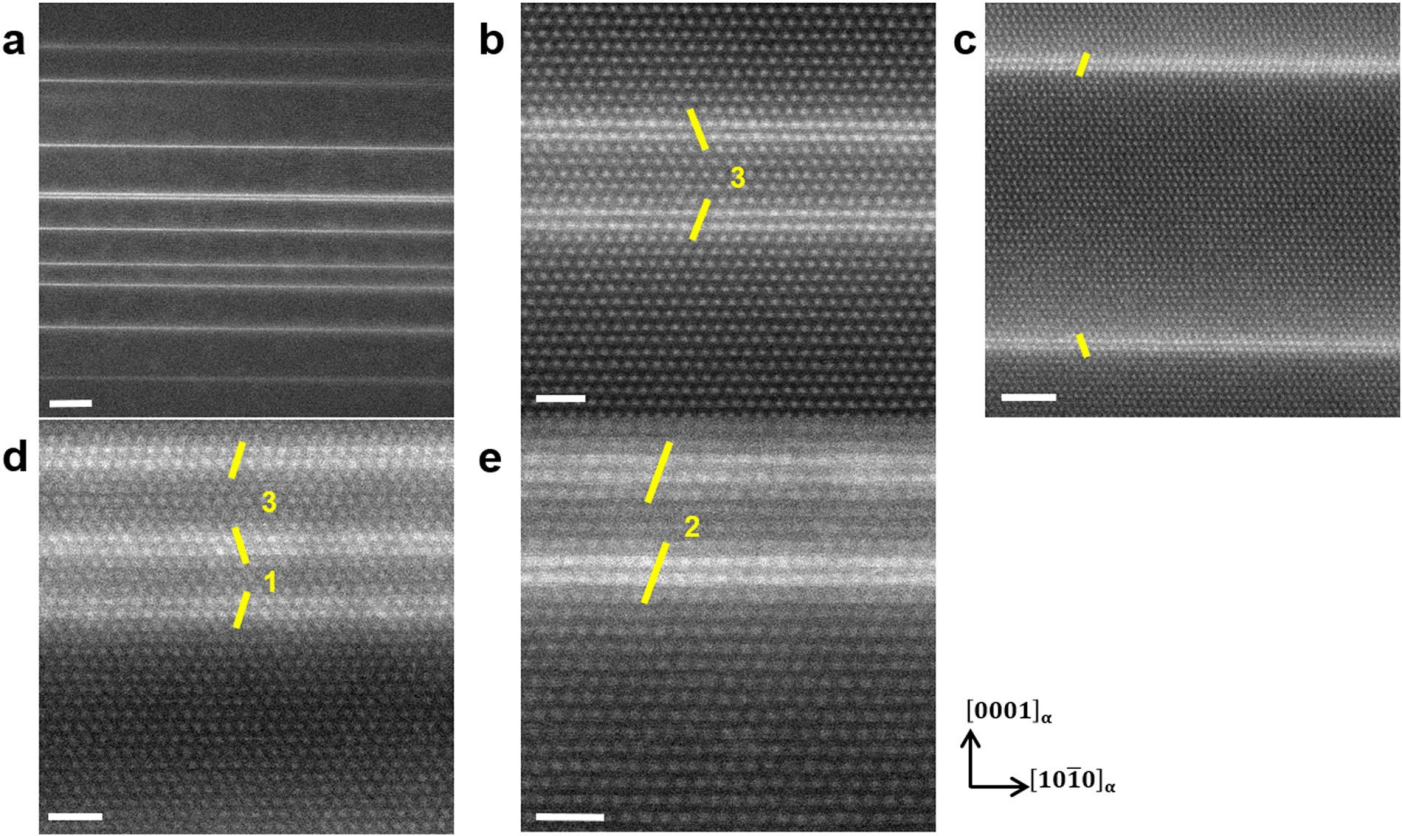
LPSO building block clusters in solution annealed Mg-1Y-0.5Zn alloy after aging at 300 °C for 10 h. HAADF-STEM images of the solution annealed Mg-1Y-0.5Zn alloy after aging at 300 °C for 10 h. The yellow lines illustrate the stacking sequence of LPSO building blocks. Beam direction is $[1\bar{2}10]$. (**a**) The microstructure after aging contains a much higher density of basal plane defects compared to the solution annealed microstructures. Scale bar width is 20 nm. (**b**) 14 H structures which are composed of two building blocks separated by three α-Mg layers are most dominantly observed after aging. Scale bar width is 5 nm. (**c**–**e**) Various metastable LPSO building block clusters such as (**c**) two single building blocks, (**d**) three building blocks separated by one and three Mg layers (“1 + 3” structure) and (**e**) two building blocks separated by two Mg layers, which can be interpreted as an intermediate state of 18 R structures. Scale bar widths are 1 nm.

Figure 4 shows HAADF-STEM micrographs of the solution annealed Mg-1Y-0.5Zn alloy after aging at 300 °C for 10 h. Compared to the solution annealed microstructures shown in Figs. 1–3, the microstructure after aging contains a much higher density of basal plane defects as shown in Fig. 4a. 14H LPSO structures are found to have mainly formed during aging at 300 °C for 10 h. 14H LPSO structures, one of the standard LPSO polytypes, are composed of two LPSO building blocks separated by three α-Mg layers (Fig. 4b) and known to be the most stable phase after long-term annealing of Mg-Y-Zn alloys^27^. On the other hand, metastable LPSO building block clusters, which have been reported to be present in the α-Mg matrix of Mg-Y-Zn alloys^27, 29, 30^, are also observed as shown in Fig. 4c–e. Figure 4c shows two single building blocks with a local fcc stacking sequence. Figure 4d presents three building blocks separated by one and three Mg layers. Following the nomenclature of our previous study^31^, the LPSO building block clusters can be called “1 + 3” structure. Figure 4e presents two building blocks separated by two Mg layers. This structure is interpreted as an intermediate state of the 18R structure^31^. No GP zones were observed after aging indicating absence or low number density of GP zones in the aged material. LPSO structures are therefore expected to grow from the GP zones during aging considering that we made no observations of GP zones and yet found a high density of LPSO structures in the aged material.

## Discussion

**Figure 5:**
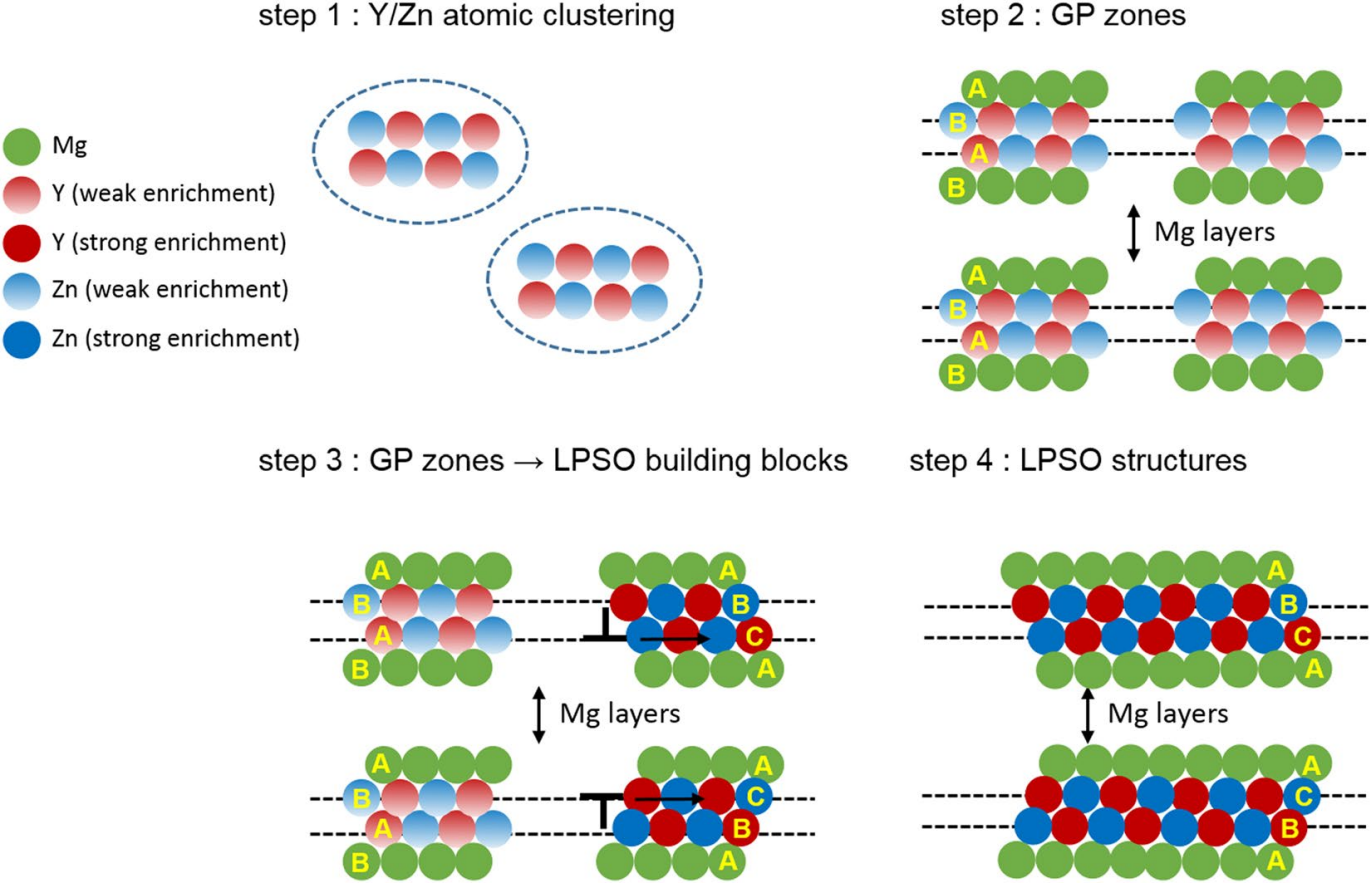
Sequential diffusional-displacive transformation mechanism. Y/Zn atomic clusters are expected to form first in the super-saturated Mg matrix (step 1) followed by the formation of Y/Zn enriched GP zones on the basal planes of the Mg matrix (step 2). Above a critical amount of Y and Zn atoms in the GP zones, LPSO building blocks are formed from the GP zones by the generation and propagation of Shockley partial dislocations (step 3). LPSO structures can be formed during annealing (step 4).

Using high resolution HAADF-STEM, we have observed the transformation of GP zones with an hcp stacking into LPSO building blocks with a local fcc stacking. This observation plays a key role in understanding the formation mechanisms of LPSO structures. Based on the present work, we propose the following formation mechanism of LPSO structures in the Mg-Y-Zn alloy (Fig. 5).

Y/Zn atomic clusters are expected to form first in the super-saturated Mg matrix (step 1). The Y/Zn atomic clustering occurs due to attractive interactions between Y and Zn atoms^2, 31^. Earlier theoretical studies^31, 32^ suggested phase separation between the Mg-rich phase (Mg matrix) and the Y and Zn enriched phase (LPSO phase) by spinodal decomposition. However, there has been no direct experimental evidence of spinodal decomposition so far in Mg-Y-Zn alloy.

The growth of Y/Zn atomic clusters leads to the formation of Y/Zn enriched GP zones on the basal planes of the Mg matrix (step 2). We did not find any clear evidence of superstructures inside the GP zones. The proposed diffusional transformation from Y/Zn atomic clusters to the GP zones is consistent with Nie *et al*.^33^ who have reported that solute clusters act as the building blocks for GP zones in binary Mg-RE alloys. Here, it is assumed that the local Y and Zn concentration in the GP zones determines the transformation into LPSO building blocks and the separation distance between the GP zones plays an important role for the formation of the different LPSO building block clusters.

Above a critical amount of Y and Zn atoms in the GP zones, LPSO building blocks are formed from the GP zones by the generation and propagation of Shockley partial dislocations (step 3). I_2_ stacking faults (stacking sequence …ABABCACA…) have a local fcc stacking sequence and are bound by $\frac{a}{3}\langle 10\bar{1}0\rangle$ - type basal <a> Shockley partial dislocations, hence being structurally identical to the observed LPSO building blocks which transformed from GP zones. Atomic scale TEM-EDXS analysis shows that the Y/Zn concentration is higher in the LPSO building blocks than in the GP zones (Fig. 3). The formation of LPSO building blocks is therefore assumed to become more favorable with increasing Y/Zn concentration. Okuda *et al*.^28^ have performed small-angle X-ray scattering measurements and suggested for an Mg_85_Y_9_Zn_6_ alloy that a cooperative shear transforms hcp-based clusters into fcc-based clusters. They^32^ also performed first-principles calculations which indicate that the transformation is energetically favorable since the energy gain associated with the transformation from Y_6_Zn_6_ + Y_2_ clusters in an hcp lattice into Y_8_Zn_6_ L1_2_ clusters sufficiently overcomes the energy loss caused by stacking-fault formation. However, considering the fact that the formation of Zn_6_Y_8_ clusters of L1_2_ type is only pronounced in heavily-alloyed Mg-Y-Zn alloys^2, 11, 12^, the proposed energetics may not be correct for the case of a lean-alloyed Mg-Y-Zn alloy. Another driving force for the formation of LPSO building blocks from GP zones might be a reduction of the basal I_2_ stacking fault energy of Mg through the addition of Y and Zn atoms. Zhu *et al*.^34^ performed TEM analysis on an Mg-Zn-Y alloy and reported a significantly reduced I_2_ stacking fault energy of an Mg-Y-Zn alloy with respect to pure Mg. Density functional theory calculations^35^ have also shown that the simultaneous addition of Y and Zn to Mg decreases the stable and unstable stacking fault energies considerably. Generally, leading and trailing Shockley partial dislocations have a repulsive interaction. The increased fault energy of the newly created stacking fault among two Shockley partial basal dislocations can be compensated by the energy reduction resulting from the decreased repulsive interaction when the two Shockley partial basal dislocations are further apart from each other. Therefore, for a low I_2_ SFE, an energetic balance is achieved for a large distance between the dissociated dislocations. In the GP zones which are assumed to have a low SFE due to the enrichment of Y/Zn atoms, the formation of wide stacking faults by the movement of Shockley partial basal dislocations could thus be energetically favorable.

After long-term annealing, the LPSO building block clusters transform to 14H structures which are thermodynamically the most stable LPSO structures^27^ (step 4). In Al-Cu alloys, coarsening of GP zones before the nucleation of thermodynamically stable CuAl_2_ phase precipitates has been observed^21, 35^. On the contrary, the GP zones observed in the present study act as nuclei for thermodynamically stable LPSO structures. The phase transformation mechanisms observed in this study can be described by a sequential diffusional-displacive transformation as shown schematically in Fig. 5.

In conclusion, we observed the direct transformation of GP zones to LPSO building blocks in a lean-alloyed Mg-Y-Zn compound. The proposed sequential phase transformation mechanism gives new insights into the phase transformation mechanisms in metallic materials. Further, the proposed mechanisms provide a framework for the design and optimization of Mg-Y-Zn alloys which show promising mechanical properties.

## Methods

An Mg-1Y-0.5Zn (wt. %) alloy was molten and cast in an induction furnace under 20 bar pressure Ar atmosphere. The cast material was solution-treated at 500 °C for 10 h, followed by air cooling. Further aging was carried out at 300 °C for 10 h, followed by water quenching. The samples for TEM were cut into discs with a diameter of 3 mm and a height of 1 mm using electric discharge machining. The discs were ground to a thickness of 150–200 µm, then twin-jet electro-polished in a solution of 5.3 g lithium chloride, 11.2 g magnesium perchlorate, 500 ml methanol and 100 ml 2-butoxy-ethanol at −30 °C. The bright-field TEM images and related selected-area diffraction patterns were obtained with a Philips CM20 microscope. High-angle annular dark-field (HAADF) imaging and energy dispersive x-ray spectroscopy (EDXS) mapping were performed on an FEI Titan G2 80–200 and a ChemiSTEM microscope equipped with a high-brightness field emission gun, a probe spherical aberration corrector and a super-X EDXS system^36^. The microscopes were operated at 200 kV. The EDXS data were processed using the software Esprit.

### Data availability

The data that support the findings of this study are available from the corresponding author upon request.
